# Obese older adults report high satisfaction and positive experiences with care

**DOI:** 10.1186/1472-6963-14-220

**Published:** 2014-05-16

**Authors:** Frank G Bottone, Shirley Musich, Shaohung S Wang, Cynthia E Hommer, Charlotte S Yeh, Kevin Hawkins

**Affiliations:** 1 Advanced Analytics, Optum, 315 E. Eisenhower Parkway, Suite 305, Ann Arbor, MI 48108, USA; 2 Current address: MCG – Formerly Milliman Care Guidelines, 901 Fifth Avenue, Suite 2000, Seattle, WA 98164, USA; 3 AARP Services, Inc., 601 E St NW, Washington, DC 20049, USA

**Keywords:** Obesity, Satisfaction, Attentiveness to care, Medicare

## Abstract

**Background:**

Obese, older adults often have multiple chronic conditions resulting in multiple health care encounters. However, their satisfaction and experiences with care are not well understood. The objective of this study was to examine the independent impact of obesity on patient satisfaction and experiences with care in adults 65 years of age and older with Medigap insurance.

**Methods:**

Surveys were mailed to 53,286 randomly chosen adults with an AARP® Medicare Supplement Insurance Plan insured by UnitedHealthcare Insurance Company (for New York residents, UnitedHealthcare Insurance Company of New York) in 10 states. Following adjustment for non-response bias, multivariate regression modeling was used to adjust for demographic, socioeconomic and health status differences to estimate the independent impact of weight on satisfaction and experiences with care. Outcome variables included four global and four composite measures of satisfaction and experiences with care.

**Results:**

21.4% of the respondents were obese. Relative to normal weight, obesity was significantly associated with higher patient satisfaction and better experiences with care in seven of the eight ratings measured.

**Conclusions:**

Obese individuals were more satisfied and had better experiences with care. Obese individuals had more office visits and discussions about nutrition, exercise and medical checks. This may have led to increased attentiveness to care, explaining the increase in satisfaction and better experiences with care. Given the high level of satisfaction and experiences with care in older, obese adults, opportunities exist for clinicians to address weight concerns in this population.

## Background

Obesity is a significant problem in much of the industrialized world, particularly in the United States [1]. Obesity is a risk factor for many chronic conditions, and is associated with increased medical [2,3] and prescription drug costs [4]. Obesity is also associated with decreased life expectancy [5] and quality of life [6]. In addition to the socioeconomic and health status differences of obese patients, these individuals often report bias in attitudes and treatment, even among health care workers [7,8]. Such biases can lead to inferior quality of care and a lack of trust in the health care system. Whether real or perceived, this is unfortunate because this is the very setting that has the most to offer in terms of education and treatment for obesity. Such missed opportunities are common across all ages and disease states. Furthermore, this can lead to differences in quality of care and ultimately satisfaction with health care services. Consequently, obese patients are more likely to “doctor shop”, which is an indicator of poor satisfaction with their care [9]. In a study of obese women with a mean age of 44 years, patients were significantly less satisfied with their obesity care and physician’s expertise compared to that of their general care and physicians’ expertise in general [10]. The authors concluded that these women were less likely to consult their physician for guidance with regard to their weight control [10]. This is consistent with a qualitative study of obese patients (mean age ~45 years) reporting that obese patients complain of their primary care physician’s knowledge and degree of engagement with regard to their weight-related issues, illustrated by the fact that over two thirds of patients said that their doctor rarely or never discussed their weight problems with them [11]. These are further indications that satisfaction with health care services is an issue in middle-aged (i.e. 30′s-50′s), obese adults.

The importance of patient surveys to assess patient satisfaction in an attempt to evaluate and ultimately improve the health care system at a local and system-wide level has been described extensively in the literature [12]. Numerous scales have been used to estimate patient satisfaction with health care services, each with their own strengths and limitations [13]. The Picker Institute Adult Inpatient Survey has been used to study satisfaction in obese adults [14]. Their results indicated that middle-aged, obese adults appeared to be less satisfied with their health care services. However, in this same study, older, obese adults seemed to report the same or significantly better (less problematic) satisfaction, relative to their younger counterparts [14]. Meanwhile, using the household component of the nationally representative, Medical Expenditure Panel Survey, the authors reported increased satisfaction with health care among older, but not younger, obese patients relative to their normal weight counterparts [15]. Therefore, age appears to be an important factor when it comes to satisfaction with services.

While it has been reported elsewhere that older obese adults report increased satisfaction using a subset analysis [14], the objective of this study was to examine the impact that obesity, as a function of body mass index (BMI), has on patient satisfaction and experiences with care in adults 65 years of age and older. The interpretation (i.e. ratings of satisfaction) of clinical encounters varies with such factors as age and health status indicating that age is an important factor in determining satisfaction, with older adults being more satisfied [16,17]. In addition, patients with chronic conditions often have increased ratings of satisfaction, at least in part, due to the fact that increased quality of care leads to more intensive care [18]. Therefore, in the present study, we attempted to estimate the independent impact of obesity on satisfaction and experiences with care in older adults, adjusting for demographic, socioeconomic and health status differences across the BMI categories. The other BMI categories were evaluated for comparison.

## Methods

### Study population

Medicare is the primary insurer for nearly all Americans 65 years or older (about 15% of the country). Medicare also covers those with certain disabilities and/or end stage renal disease regardless of age. Among those with Medicare coverage (an estimated 52 million Americans in 2013), 73% have original fee-for-service coverage; whereas, the rest have a Medicare Advantage plan (a type of managed care) [19]. Of those with original fee-for-service coverage, approximately 9 million people purchased Medigap insurance plans to defray the out-of-pocket expenses from copayments, coinsurance and deductibles that Medicare does not cover in entirety [20]. As part of an effort to understand the needs of this population and to assess patient perceptions of satisfaction in this population, a survey (detailed below) was mailed to a randomly selected sample of 53,286 Medicare beneficiaries in ten states between 2009 and 2011. Eligible participants were 65 years of age or older with a Medicare Supplement Insurance Plan insured by UnitedHealthcare Insurance Company (for New York residents, UnitedHealthcare Insurance Company of New York).

In addition to the standard questions, additional questions about their supplement insurance plan, height and weight were included for analysis. Eligible survey respondents included those with available height and weight information required to calculate body mass index (BMI). Data from survey respondents were divided into the following standard BMI categories based on their self-reported height and weight: underweight (BMI at or below 18.5), normal-weight (BMI 18.6–24.9), overweight (BMI 25–29.9) and obese (BMI 30 or greater) [21]. Insufficient numbers were available (i.e. <2% were morbidly obese) to allow for analysis by obesity class (i.e. obesity class I-III).

### Data collection

Data for this study was collected from a modified version of the fee-for-service Consumer Assessment of Healthcare Providers and Systems (CAHPS) survey. The CAHPS survey was designed to assess consumer satisfaction and experiences with health care services [22,23]. The survey is administered to Medicare Advantage and other plan recipients annually as a measure of patient satisfaction. Therefore, identifying differences in patient populations using CAHPS can be a valuable tool to identify issues with patient satisfaction thereby increasing awareness and providing opportunity for improvements. Medicare is a government entitlement program, and is the primary form of medical insurance for adults 65 or older in the United States. As the government continues to emphasize quality of care and satisfaction with services while aligning payment for services with quality of care, a greater understanding of these factors might help lead to greater satisfaction, especially in those with the greatest need (i.e. those with chronic conditions). Blinded survey response data was collected following random sampling of eligible participants with oversampling for minorities. Data was analyzed using SAS Version 9.2 (Cary, NC, USA). The survey response rate was 37.7%. This response rate is typical of large-scale, single-wave mailings.

### Outcomes of interest

The outcomes of interest included responses to questions on patient satisfaction and experiences with care contained in the survey. Specifically, the survey included validated global and composite rating scale scores [24]. Global ratings included questions on satisfaction with their personal doctor, specialist, all health care and Medicare Supplement Insurance. Global satisfaction ratings responses ranged from 0–10. Composite ratings included two or more questions on experiences with care combined into broad categories such as doctor communication, chronic condition management, care coordination and access to care. Composite ratings were based on four-level (never, sometimes, usually or always) individual question responses [22,24]. Global and composite scores were transformed to a 0–100 scale for ease of comparison as reported elsewhere [25]. The global and composite ratings scores were then dichotomized into ninety or greater or below in an effort to create a binomial distribution and are represented as odds ratios dictated by the binomial distribution based on the distribution of the data and as reported elsewhere [26]. A score was calculated if at least 50% of the items in the scale were completed (this is commonly referred to as the “half-scale” rule) as is common with survey data. The outcomes of interest with specific questions from the survey are detailed in Table 1.

**Table 1:** Questions used for the global ratings and composite scores

**Outcome variable**^**a**^	**Category**	**Survey question (s)**
Global ratings (satisfaction)	Personal doctor	Using any number from 0 to 10, where 0 is the worst personal doctor possible and 10 is the best personal doctor possible, what number would you use to rate your personal doctor?
	Specialist	We want to know your rating of the specialist you saw most often in the last 6 months. Using any number from 0 to 10, where 0 is the worst specialist possible and 10 is the best specialist possible, what number would you use to rate the specialist?
	Health care	Using any number from 0 to 10, where 0 is the worst health care possible and 10 is the best health care possible, what number would you use to rate all of your health care in the last 6 months?
	Supplement plan	Using any number from 0 to 10, where 0 is the worst Medicare supplement insurance plan possible and 10 is the best health plan possible, what number would you use to rate the AARP Medicare Supplement Insurance Plan?
Composite ratings (experiences with care)	Doctor communication	In the last 6 months, how often did your personal doctor explain things in a way that was easy to understand?
		In the last 6 months, how often did your personal doctor listen carefully to you?
		In the last 6 months, how often did your personal doctor show respect for what you had to say?
		In the last 6 months, how often did your personal doctor spend enough time with you?
	Chronic condition management	In the last 6 months, did your personal doctor give you clear instructions about how to manage your health condition?
		In the last 6 months, did your personal doctor work with you to set personal goals for managing your health condition?
	Care coordination	In the last 6 months, how often did your personal doctor seem informed and up to date about the care you got?
		In the last 6 month, when your personal doctor sent you for tests, how often did someone from the doctor’s office follow-up to give you test results?
	Access to care	In the last 6 months, when you needed care right away, how often did you get care as soon as you thought you needed?
		In the last 6 months, not counting the times you needed care right away, how often did you get an appointment for your health care at a doctor’s office as soon as you thought you needed it?
		In the last 6 months, how often was it easy to get the care, tests or treatment you thought you needed?
		In the last 6 months, how often was it easy to get appointments with specialists?

^a^The two main outcomes (global and composite ratings) included four measures of satisfaction and four measures of experiences with care, respectively.

### Covariates

To estimate the independent impact of each BMI category on patient satisfaction and experience with care, outcome variables were adjusted for various demographic, socioeconomic and health status differences. Demographic and socioeconomic questions on the CAHPS included age, gender, race, marital status, living arrangements and education level. Information on their state of residence and income were geocoded from their zip code as reported elsewhere [27]. Questions about health status included those on commonly treated health conditions (e.g., diabetes, hypertension, respiratory disorders) as well as physical and mental health status.

### Statistical analysis

Due to the relatively low response rate seen with single-wave mailing without phone reminders, the results were adjusted for survey non-response bias to increase the generalizability using the entire survey population (about 50,000 surveys were mailed out). The first analysis was descriptive, and categorized sample respondents by demographics, socioeconomics and clinical characteristics and compared respondents in underweight, normal, overweight and obese BMI categories. Chi-square and Student t-tests were used to test for differences in categorical and continuous variables, respectively. The second analysis adjusted the data for survey non-response bias using the available covariates (i.e. gender, age, minority status and state of residence). Subsequently, multivariate logistic regression techniques were used to estimate the independent impact of each BMI category on the outcomes of interest, controlling for patient demographic, socioeconomic and other health status differences detailed above across the weight groups. All analyses were performed using SAS software (version 9.1; Cary, NC). A sensitivity analysis was also performed to estimate the impact of excluding new members, which might positively bias these results, however, this had little effect on the outcome measures; therefore, these respondents were included in the analysis (data not shown).

To address attentiveness to care, we analyzed responses from the following two standard CAHPS survey questions to look for differences among the groups regarding discussions with physicians on various lifestyle topics relevant to weight management, which served as a proxy for attentiveness to care. The questions were as follows: “In the last 6 months, did your personal doctor or any other provider ask you to do the following to help manage this health condition?” and “In the last 6 months, how many times did you visit your personal doctor to get care for yourself?” As frequency of visits could potentially bias satisfaction, visit frequency was controlled for in these analyses using self-reported number of visits to their primary care physician [28].

### Ethical considerations

This retrospective analysis of survey data was reviewed and approved by the New England Institutional Review Board and was granted waiver of informed consent. This study was performed in accordance with the principles outlined in the Declaration of Helsinki [29] and in compliance with the “Protection of Human Subjects and Animals in Research” as described in the recommendations of the International Committee of Medical Journal Editors [30].

## Results

### Sample characteristics

**Figure 1:**
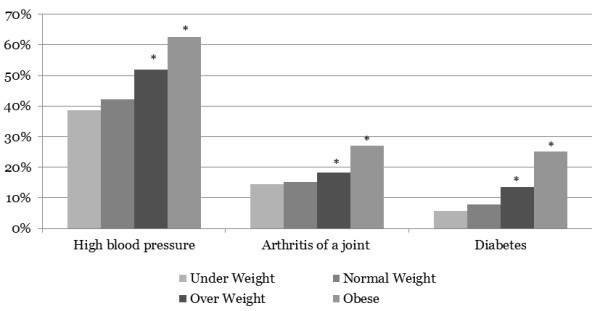
**Prevalence of select chronic conditions.** Note: Percentages are before adjusting for survey non-response bias and the regression analyses. *Denotes statistically significant at p < 0.01.

The demographic, socioeconomic and health status characteristics of the eligible survey respondents are detailed in Table 2 according to BMI category. After excluding those without BMI information (9% of respondents), there were 18,192 eligible respondents included in this analysis. Respondents were primarily female, White, living in a metropolitan area with most in the 70–74 years of age group. The respondents had the following BMI categories: 2.8% were underweight, 38.6% had a normal BMI, 37.2% were overweight and 21.4% were obese. The most common comorbid condition in the obese category was high blood pressure, followed by arthritis of a joint and diabetes, each of which increased with increasing BMI (Figure 1). Low back pain and respiratory disease were also highest in the obese category, but did not show the same trend. Based on the demographic information, relative to the normal weight group, those in the obese category were more likely to have high blood pressure, arthritis of a joint, respiratory disease, diabetes and other comorbid conditions. They were less likely to report excellent or very good general physical health, problems performing activities of daily living and more likely to visit their personal doctor in the past six months.

**Table 2:** Characteristics of the study population before adjustments for survey non-response bias

	**Before adjusting for survey non-response**				
	**Total population**	**Underweight**	**Normal weight**	**Overweight**	**Obese**
**Participants (%)**^**a**^	100%	2.8%	38.6%	37.2%	21.4%
Participants (n)	18,192	516	7,018	6,765	3,893
**Mean age**	77.2	80.8	78.7	76.8	74.9
**Gender**					
Male	39.4%	21.1%	32.9%	47.9%	38.8%
Female	60.6%	78.9%	67.1%	52.1%	61.2%
**Age**					
65 - 69	18.9%	10.5%	15.2%	19.1%	26.5%
70 - 74	24.3%	17.6%	21.1%	25.4%	29.0%
75 - 79	18.1%	12.8%	17.1%	19.0%	19.1%
80 - 84	18.3%	25.2%	19.3%	18.5%	15.3%
85 plus	20.3%	33.9%	27.2%	18.0%	10.2%
**Income**^**b**^					
High	46.3%	42.6%	47.0%	47.1%	44.0%
Upper medium	23.5%	23.5%	23.5%	23.3%	23.7%
Lower medium	16.2%	19.4%	15.8%	15.9%	17.0%
Low or missing	14.1%	14.5%	13.8%	13.7%	15.3%
**Race**					
White	90.8%	85.5%	90.2%	91.5%	91.3%
African American	4.4%	4.8%	3.5%	4.5%	5.7%
Other	4.1%	9.1%	5.5%	3.3%	2.3%
**Education attainment**					
High school graduate or less	41.2%	47.5%	39.5%	40.8%	44.4%
Some college or 2-year degree	27.7%	25.2%	26.8%	27.3%	30.1%
4 or more year college	30.2%	26.6%	32.7%	31.1%	24.8%
**Living arrangement**					
Personal home or apartment	92.1%	85.9%	91.1%	93.1%	93.0%
Other (Long term care, assisted living facility or Other)	5.1%	10.3%	5.8%	4.4%	4.3%
**Need someone’s help to complete this survey**					
Yes	9.8%	15.5%	10.7%	8.7%	9.4%
No	88.2%	82.2%	87.2%	89.3%	88.9%
**Comorbidities**					
High blood pressure	50.0%	38.8%	42.1%	52.0%	62.5%
Arthritis of a joint	18.9%	14.5%	15.3%	18.2%	27.1%
Diabetes	13.6%	5.8%	7.9%	13.5%	25.1%
Low back pain	11.3%	9.9%	10.2%	10.2%	15.4%
Other heart conditions such as heart valves or rhythm	11.0%	13.6%	11.0%	10.8%	11.3%
Osteoporosis	10.6%	16.1%	13.8%	8.5%	7.7%
Respiratory disease^c^	10.4%	14.0%	9.8%	9.2%	13.3%
Depression	7.0%	6.8%	6.2%	6.5%	9.5%
Chest pain or coronary artery disease	6.8%	6.0%	5.5%	7.3%	8.3%
Digestive or bowel problems^d^	6.4%	8.5%	7.1%	5.6%	6.3%
Any cancer	5.5%	6.6%	5.2%	5.6%	5.7%
Weak heart or congestive heart failure	4.1%	5.8%	3.8%	3.5%	5.4%
Stroke	2.0%	2.1%	1.8%	2.1%	2.2%
Heart attack	1.7%	1.0%	1.4%	1.7%	2.1%
**General physical health**					
Excellent or very good	40.9%	32.6%	45.9%	42.9%	29.5%
Good	37.2%	34.3%	33.9%	38.2%	42.0%
Fair or poor	18.3%	28.5%	16.7%	15.3%	24.9%
**General mental health**					
Excellent, very good or good	89.6%	83.1%	88.9%	91.0%	89.4%
Fair or poor	7.3%	12.4%	8.0%	5.9%	7.6%
**Activities of daily living**					
0 - 1	72.5%	65.1%	76.0%	75.5%	62.2%
2 - 3	13.8%	14.9%	10.7%	12.6%	21.3%
4 - 5	4.8%	6.0%	4.4%	4.0%	6.9%
6	4.0%	6.8%	4.0%	3.5%	4.6%
Activities of daily living (Summary Score)	10.8	10.4	10.9	10.9	10.5

^a^For brevity, the percentage of those with missing values for each category are not shown (unless otherwise shown) but were included in the analysis, thus categories may not total 100%. The analysis also adjusted for self-reported number of visits to their personal doctor, year surveyed, state of residence, smoking status and a proxy for health literacy (data not shown for brevity).

^b^Income was geocoded based on respondent’s zipcode.

^c^Respiratory disease included self-reported emphysema, asthma and chronic obstructive pulmonary disease (COPD).

^d^Digestive disorders included self-reported Crohn’s disease, ulcerative colitis or inflammatory bowel disease.

### Satisfaction and experiences with care

Global ratings of satisfaction and composite ratings on experiences with care were estimated following adjustment for non-response bias and various patient demographic, socioeconomic and health status differences as detailed in the methods. This allowed us to estimate the independent impact of each BMI category on satisfaction and experiences with care. Following those adjustments, obese individuals were 9%-22% more likely (i.e. odds ratios of 1.09 to 1.22) to report higher satisfaction (i.e. global rating score of 90 or more) in each of the categories measured as detailed in Table 3. Meanwhile, obese individuals were 12%-21% more likely (i.e. odds ratios of 1.12 to 1.21) to report better experiences with care (i.e. global rating score of 90 or more) in three of the four categories measured (Table 3).

**Table 3:** Patient satisfaction and experiences with care following multivariate regression to control for various demographic, socioeconomic and health status differences

	**BMI category**	**Odds ratio estimate**^**a**^	**Lower C.I.**	**Upper C.I.**	**p-value**
**Global ratings (Satisfaction)**					
Global satisfaction rating for personal doctor	Underweight	0.84	0.73	0.96	0.01
	Overweight	1.03	0.98	1.09	0.23
	Obese	1.22	1.13	1.31	<0.0001
Global satisfaction rating for specialist (9 or higher out of 10)	Underweight	0.98	0.83	1.16	0.81
	Overweight	1.02	0.96	1.08	0.61
	Obese	1.13	1.04	1.23	<0.01
Global satisfaction rating for all health care (9 or higher out of 10)	Underweight	1.03	0.91	1.16	0.66
	Overweight	1.06	1.01	1.11	0.01
	Obesity	1.19	1.12	1.27	<0.0001
Global satisfaction rating for AARP supplement insurance (9 or higher out of 10)	Underweight	0.98	0.86	1.11	0.74
	Overweight	1.01	0.96	1.05	0.76
	Obese	1.09	1.02	1.16	0.01
**Composite ratings (Experiences with care)**					
Personal doctor communication: Personal doctor explained things in a way that was easy to understand, listen carefully to you, show respect for what you had to say, and spend enough time with you	Underweight	0.88	0.77	1.01	0.07
	Overweight	1.06	1.01	1.11	0.03
	Obese	1.21	1.13	1.29	<0.0001
Chronic condition management: Personal doctor gave clear instructions about how to manage your health condition, and work with you to set personal goals for managing your health condition	Underweight	0.93	0.80	1.07	0.29
	Overweight	0.89	0.85	0.94	<0.0001
	Obese	0.97	0.91	1.04	0.41
Composite patient experience scores for care coordination is 90 or higher out of 100	Underweight	0.95	0.83	1.09	0.44
	Overweight	1.08	1.03	1.14	0.001
	Obese	1.2	1.12	1.28	<0.0001
Composite patient experience scores for access to the care is 90 or higher out of 100	Underweight	0.9	0.79	1.03	0.13
	Overweight	1.04	0.99	1.09	0.11
	Obese	1.12	1.06	1.20	<0.001

^a^Relative to normal weight. Results of the main outcome measures are after adjustment for survey non-response bias and regression analysis as indicated in Table 2.

### Questions relating to attentiveness to care

Table 4 details the unadjusted responses to two questions, “In the last 6 months, did your personal doctor or any other provider ask you to do the following to help manage this health condition?” and “In the last 6 months, how many times did you visit your personal doctor to get care for yourself?” As a result, compared to normal weight individuals, obese individuals were 16.6 percentage points higher (i.e. 54% increase) in reporting that their doctor recommended checking their weight regularly and 16.4 percentage points higher (i.e. 58% increase) in reporting being recommended to check their blood sugar regularly. The results from the question on visit frequency are illustrated in Table 4. Obese individuals were more likely to have three or more visits to their personal doctor in the past 6 months (i.e. a 26% increase).

**Table 4:** Questions relating to attentiveness to care

	**Underweight**	**Normal weight**^**a**^	**Overweight**	**Obese**
*In the last 6 months, did your personal doctor or any other provider ask you to do the following to help manage this health condition*				
**Check your weight regularly**	14.0%	14.0%	20.3%**	30.6%**
**Check your blood sugar regularly**	9.3%*	11.7%	17.2%**	28.1%**
**Exercise or do specific physical activities**	27.5%**	34.5%	41.7%**	49.0%**
Check your blood pressure regularly	31.0%	31.2%	36.9%**	41.1%**
Avoid particular foods	14.9%*	19.3%	23.6%**	29.1%**
Eat particular foods	11.1%*	14.9%	16.5%**	21.2%**
Go to a particular group or class	2.3%	2.4%	2.5%	3.9%**
Read a booklet or watch a video about this condition	2.1%	2.2%	2.4%	2.8%*
Take prescription medicine	64.5%	63.9%	67.6%**	74.1%**
*In the last 6 months, how many times did you visit your personal doctor to get care for yourself?*				
None	14.3%	14.5%	12.1%**	9.9%**
1	27.7%*	31.3%	30.6%	27.2%**
2	24.8%	23.7%	26.6%**	28.2%**
3 or more	23.1%	23.4%	24.9%*	29.6%**

^a^Results are prior to adjustments for survey non-response bias. Visit frequency was adjusted for in the outcomes analyses.

Results were similar after adjustments but are not shown for brevity.

Statistically significant according to Chi-square test relative to normal weight group at *p < 0.05 and **p < 001, respectively.

## Discussion

The present study examines the impact of obesity on satisfaction and experiences with care in adults 65 years of age and older with Medigap insurance. Using the fee-for-service CAHPS survey instrument, we estimated the independent impact of weight on satisfaction and experiences with care, while controlling for various demographic, socioeconomic and health status differences. In this study, obese individuals reported higher ratings of satisfaction and experiences with care (i.e. global rating score of 90 or more) for most of the measures tested compared to their normal weight counterparts.

Satisfaction is often associated with quality of care; however, it is important to draw conclusions cautiously, as there is only a moderate association between these two matters indicating other factors are involved. Preventive services are a major factor when considering quality of care. Quality of care is generally high in obese individuals because they often receive the same or even a greater number of many preventive screenings due to the presence of comorbid conditions, leading to higher quality of care scores. In a recent study by Littman et al., obese individuals of varying ages received more of the more common preventive services (i.e. vaccinations, cholesterol, HIV testing) but less of other more involved procedures (i.e. colorectal, cervical and breast cancer screenings) [31]. This is in alignment with numerous other studies [32-35].

Despite receiving the same or more of the less complex screening procedures, obese individuals often receive fewer of the more complex screenings, which may, at least in part, contribute to the increased mortality (yet higher quality of care) seen in older obese individuals [36]. This premise is supported by our findings that the mean age of the obese individuals in this study was younger than the mean age of the normal weight group (i.e. 74.9 versus 78.7 years, respectively), likely a result of increased mortality. Additionally, the percentage of obese individuals 65 or older in our study was less than that of the United States 65 or older population at large (21% compared to nearly 35%, respectively) based on clinician-obtained data (as opposed to self-reported described herein) from the National Health and Nutrition Examination Survey (NHANES) [37]. Therefore, some, but not all of these differences are due to the self-reported nature of the present study. Additionally, there are socioeconomic differences in our population compared to that of the national sample, which likely led to some of these differences.

The present study is unique in that it deals with adults 65 years or older. It has been reported elsewhere that older (but not younger), obese individuals are more satisfied with care [38,15]. In society, there is a stigma often associated with obesity resulting in various negative consequences ranging from disparities in gaining employment, social rejection, negative stereotypes and reduced access to health care (all of which are associated with poorer health outcomes) [39]. This stigmatism is especially true of younger, obese adults, which may explain the differences seen on older, obese adults [40-44]. Therefore, age is an important factor affecting satisfaction in obese individuals, explaining the discrepancy between studies on satisfaction in obese individuals. This may help explain the differences in results of studies on obesity and satisfaction seen across different ages.

Given the fact that younger, obese adults often report lower satisfaction, another possible explanation for the increased satisfaction in older, obese adults is attentive care evident by the fact that they are being talked to about their chronic conditions, regular health checks, diet and exercise as reported herein [15]. Attentiveness to care often comes about through more frequent and intense visits, as is seen in those with chronic conditions. For example, it has been demonstrated that patients with complex medical conditions (such as older adults in general) receive more attentive care for their conditions, which correlates with increased satisfaction [28].

Increased satisfaction is seen in patients with chronic conditions that require more intense management. Using the CAHPS survey and similar analytical methods, it was demonstrated that patients with end stage renal disease experience greater satisfaction and better experiences with care [25]. Increased attentiveness is evident in our study by the self-reported, unadjusted increased number of self-reported discussions around nutrition, exercise and medical checks (e.g. glucose, blood pressure and weight) with their physician. Similarly, obese individuals were more likely to have three or more visits to their personal doctor in the past 6 months in this study. While this was controlled for in the analysis, some impact on attentiveness may remain.

The limitations of our study include the reliance upon self-reported height and weight data, which may slightly underestimate BMI [45]. That said, the use of BMI is sufficiently accurate to categorize individuals into the appropriate weight categories. Although we adjusted for non-response bias, other limitations include the relatively low response rate of this study, and the fact that chronic conditions were self-reported. Additionally, the study sample consisted only of beneficiaries 65 years of age or greater enrolled in an AARP Medicare Supplement Insurance plan, thus, may not be generalizable to all Medicare enrollees. Lastly, in addition to attentiveness to care, the increased satisfaction in these individuals might be explained by other unforeseen/uncontrolled factors such as other comorbid conditions, familiarity with the health care system or even favorable or misconstrued bias associated with obese individuals [7,8].

In the coming years, the number of obese adults that utilize Medicare for their primary health insurance is expected to rise [46]. As various efforts are undertaken to ameliorate the obesity epidemic in younger and older adults alike, it is critical to maintain a high level of satisfaction in this population while continuing to deliver the proper messages about lifestyle and health risks. Improved outcomes might be achievable by greater promotion and utilization of currently available and reimbursed services such as Medicare’s coverage for intensive behavioral therapy in appropriate patients [47]. From a population health perspective, programs such as the various “Maintain, Don’t Gain” weight management campaigns (i.e. maintaining your current health risk level) or similar programs that promote physical activity to maintain weight should be considered in appropriate populations [48]. Such programs are realistic in that they recognize weight maintenance in older adults can be considered a success due to their reduced caloric needs as we age in an environment where there is an abundance of high calorie, low cost options [49].

Therefore, consistent with our hypothesis, the present study adds to the literature that obesity, independent of other factors controlled for in this study, has a positive impact on satisfaction and positive experiences with care in older adults, likely due to increased attentiveness to care. This explanation is reasonable in that many aspects of quality of care appear to be as good or greater in adults with obesity-related syndromes [50]. That said, while physicians appeared to provide more attentive care to obese individuals (i.e. were more likely to discuss topics such as eating and exercise while recommending regular checking of health measures) the degree to which they discussed obesity is unknown. While conversations about weight management might be difficult for members of the clinical team (physician, nurse, case manager, health coach), engaging patients with these conversations about weight loss and increased exercise while managing realistic expectations in an attempt to achieve positive outcomes appears warranted [11]. Therefore, programs that take a more holistic approach to managing health by focusing on achieving optimal quality of care and living a healthy lifestyle are needed.

## Conclusions

In the present study, obese individuals were more satisfied and had better experiences with care. Obese individuals had more office visits and discussions about nutrition, exercise and medical checks. This may have led to increased attentiveness to care, explaining the increase in satisfaction and better experiences with care seen in obese individuals. The present study adds to our understanding of how obese older adults perceive care, while informing practitioners that such patients can be pushed harder to take an active role in their health.

## Abbreviations

BMI: Body mass index; CAHPS: Consumer assessment of healthcare providers and systems; NHANES: National Health and Nutrition Examination Survey; COPD: Chronic obstructive pulmonary disease.

## Competing interests

Kevin Hawkins and Cindy Hommer have stock options with UnitedHealth Group. Authors are paid employees of their respective organizations listed on the title page.

## Authors’ contributions

FGB, SM, SSW, CEH, CSY and KH contributed to the design, analysis and writing of this manuscript. All authors read and approved the final manuscript.

## Pre-publication history

The pre-publication history for this paper can be accessed here:

http://www.biomedcentral.com/1472-6963/14/220/prepub
